# Mutagenic and Cytotoxicity LQB 123 Profile: Safety and Tripanocidal Effect of a Phenyl-t-Butylnitrone Derivative

**DOI:** 10.1155/2017/2483652

**Published:** 2017-02-15

**Authors:** Mauricio Peixoto Cupello, Francis Monique Saraiva, Pedro Ippolito, Andréia da Silva Fernandes, Rubem Figueiredo Sadoko Menna-Barreto, Debora de Sousa dos Santos Costa, Jessica Isis Oliveira Paula, Paulo Roberto Ribeiro Costa, Natália Pereira Nogueira, Israel Felzenswalb, Ayres Guimarães Dias, Marcia Cristina Paes

**Affiliations:** ^1^Laboratório de Interação Tripanossomatídeos e Vetores, Departamento de Bioquímica, Instituto de Biologia Roberto Alcantara Gomes, Universidade do Estado do Rio de Janeiro, Rio de Janeiro, RJ, Brazil; ^2^Unidade de Desenvolvimento Tecnológico (UDT) de Triagem de Compostos Químicos para Doenças Negligenciadas com Ênfase Farmacológica contra a Doença de Chagas, Universidade do Estado do Rio de Janeiro, Rio de Janeiro, RJ, Brazil; ^3^Laboratório de Mutagênese Ambiental LABMUT, Departamento de Biofísica e Biometria, Instituto de Biologia Roberto Alcantara Gomes, Universidade do Estado do Rio de Janeiro, Rio de Janeiro, RJ, Brazil; ^4^Laboratório de Biologia Celular, IOC-FIOCRUZ, Rio de Janeiro, RJ, Brazil; ^5^Departamento de Química Orgânica, Universidade do Estado do Rio de Janeiro, Rio de Janeiro, RJ, Brazil; ^6^Laboratório de Química Bioorgânica, NPPN, Universidade Federal do Rio de Janeiro, Rio de Janeiro, RJ, Brazil; ^7^Instituto Nacional de Ciência e Tecnologia, Entomologia Molecular (INCT-EM), Rio de Janeiro, RJ, Brazil

## Abstract

The therapeutic options for Chagas disease are limited and its treatment presents a number of drawbacks including toxicity, drug resistance, and insufficient effectiveness against the chronic stage of the disease. Therefore, new therapeutical options are mandatory. In the present work, we evaluated the effect of a phenyl-*tert*-butylnitrone (PBN) derivate, LQB 123, against* Trypanosoma cruzi* forms. LQB 123 presented a trypanocidal effect against bloodstream trypomastigotes (IC_50_ = 259.4 ± 6.1 *μ*M) and intracellular amastigotes infecting peritoneal macrophages (IC_50_ = 188.2 ± 47.5 *μ*M), with no harmful effects upon the mammalian cells (CC_50_ values greater than 4 mM), resulting in a high selectivity index (CC_50_/IC_50_ > 20). Additionally, metacyclic trypomastigotes submitted to LQB 123 presented an IC_50_ of about 191.8 ± 10.5 *μ*M and epimastigotes forms incubated with different concentrations of LQB 123 presented an inhibition of parasite growth with an IC_50_ of 255.1 ± 3.6 *μ*M. Finally, we investigated the mutagenic potential of the nitrone by the* Salmonella*/microsome assay and observed no induction of mutagenicity even in concentrations as high as 33000 *μ*M. Taken together, these results present a nonmutagenic compound, with trypanocidal activity against all relevant forms of* T. cruzi*, offering new insights into CD treatment suggesting additional in vivo tests.

## 1. Introduction

Chagas disease, recognized by WHO as one of the world most important neglected tropical diseases, is a relevant socioeconomic problem in many countries [1]; it infects approximately 7 million people worldwide and is the most relevant parasitic killer in the Americas, where it is endemic [2].* T. cruzi* is an obligate intracellular parasite with a complex life cycle that alternates between vertebrate host and invertebrate haematophagous triatomine insect and involves proliferative (amastigotes, epimastigotes) and nonproliferative (metacyclic, culture, or bloodstream trypomastigotes) stages [3]. Today, the epidemiological profile has changed due to migratory movements, which have led to both the urbanization and globalization of the disease [4, 5] since this illness is now observed in cities in the North of Americas and Europe where immigrants of endemic countries live nowadays [6, 7]. From the economic perspective, the premature mortality and significant disability caused by CD result in a large financial impact, extending over the public healthcare system, intensifying the need for organs transplant and preventive measures such as blood screening, and monitoring the infected patients with this disease [5, 8, 9]. Today, less than 1% of people infected with* T. cruzi* have access to diagnosis and treatment [10].

Forty years after the first drugs were introduced, the chemotherapy against the etiological agent of Chagas disease remains unsatisfactory. Benznidazole (BZ) and Nifurtimox failed to control the illness in two aspects: limited efficacy for treating the chronic phase of the infection, need to be used in long-term therapy, and its high systemic toxicity including skin rashes, nausea, and kidney and liver failure. Recently, a five-year clinical trial using benznidazole (BZ) against the cardiac form of Chagas disease (BENEFIT clinical trial) demonstrated that most of BZ treated patients presented undetectable parasite loads, however, with no improvement on the cardiac disease [11, 12]. Additionally, Nifurtimox can also cause seizures and other nervous-system disorders [4]. Thus, the search for a more effective therapeutic and less toxic conduct becomes imperative. The general chemical structure of nitrone is X-CH=NO-Y, where “X” is a phenyl group and “Y” is a* tert*-butyl group (PBN). This structure has been extensively studied in many experimental and biochemical systems over the past 30 years. These nitrones were initially used in biological systems, primarily due to their “spin-trap” free radicals ability [13]. Later, it was demonstrated that nitrones have low toxicity and are also suited for multiple dose regimens. Accordingly, PBN has been shown to be a relatively safe drug, even at high concentration. Despite that, the assessment of effectiveness against risk of adverse outcomes is imperative to support a clinical use [14].

There are good evidences that PBN is effective against a variety of microorganisms such as trypanosomatids. The leishmanicidal activity was also observed for the PBN derivative, C-(6-methyl-4-oxo-4H-1-benzopyran-3-yl)-N-(p-tolyl), known as NP1, which protected infected rats against the parasite by the modulation of the host immune system [15]. In 2010, Chagasic rats with cardiomyopathy, treated with an association of benznidazole and PBN, diminished oxidative damage, parasite persistence, and inflammatory pathology harmful damage. However, the same study showed that mice treated only with PBN presented no decline in parasite persistence, pointing out the need of chemical modifications in the PBN molecule in order to increase its trypanocidal effect [16].

Additionally, we demonstrated for the first time the protective activity of less expensive* N*-methyl nitrones against microvascular damage induced by occlusion-induced ischemia-reperfusion in the hamster cheek pouch preparation. LQB-123 was as active as or more active than *α*-tocopherol, shark cartilage, and fish oil, used as references [17, 18] Thereafter,* O*-isoprenylated-*N*-methylarylnitrones analogues have been synthetized and the antineoplastic effects on human cancer cell lines (Jurkat and leukemia) were demonstrated [19].

Here, we evaluated the activity of a novel PBN derivate, the LQB 123, against various biological forms of* Trypanosoma cruzi*. Our results demonstrate the trypanocidal effect of the LQB-123 against both proliferative and infective forms of* T. cruzi* with no inhibition of mammalian cell viability, suggesting this compound as a promising candidate for in vivo studies.

## 2. Materials and Methods

### 2.1. Drug Dilution

LQB 123 was synthesized as described in Kim et al. [17] and Dias et al. [18]. Stock solutions of LQB-123 were prepared in dimethyl sulfoxide (DMSO, MERCK-USA), with the final concentration of the solvent used in the experiments never exceeding 1%.

### 2.2. LQB 123 Activity upon Epimastigotes

*Trypanosoma cruzi* Y strain was provided by the Trypanosomatid Collection of the Oswaldo Cruz Institute, Fiocruz, Brazil. Epimastigotes (1 × 10^6^ parasites/mL) in the exponential phase of growth (96 h) were maintained and incubated in the presence of brain-heart infusion medium (BHI, BD Bacto, USA) supplemented with 30 *μ*M heme (Frontier Scientific, Utah, USA) and 10% fetal calf serum (FCS, Vitrocell, Campinas, Brazil) at 28°C. Then, increasing concentrations of LQB 123 (75–1200 *μ*M) were added or not to the epimastigotes in 96-well plates for 48 h. The inhibitory concentration responsible for 50% reduction in cell viability (IC_50_) was obtained after 48 h of treatment and determined by regression analysis of the data.

### 2.3. LQB 123 Activity upon Metacyclic Trypomastigotes

For in vitro differentiation, we performed the protocol determined by Contreras et al. [20] with minor modifications. Briefly, epimastigotes Dm28c were grown at 28°C for 7 days in BHI supplemented with 30 *μ*M heme and 10% FCS. Parasite growth was monitored by cell counting in a Neubauer chamber. Epimastigotes were harvested by centrifugation and then incubated in triatomine artificial urine (TAU) medium (190 mM NaCl, 17 mM KCl, 2 mM MgCl_2_, 2 mM CaCl_2_, 8 mM phosphate buffer pH 6.0) at a density of 5 × 10^8^ cells/mL for 2 h at 28°C. Next, epimastigotes were diluted 1 : 100 (5 × 10^6^ cells/mL) in TAU3AAG medium (TAU supplemented with 10 mM L-proline, 50 mM L-sodium glutamate, 2 mM L-sodium aspartate, and 10 mM D-glucose). After 96 h, culture supernatants containing metacyclic trypomastigotes were incubated in the absence or in the presence of 150 *μ*M, 300 *μ*M, 600 *μ*M, and 1200 *μ*M LQB 123 for 24 h. The parasites viability was measured by cell counting in a Neubauer chamber. The inhibitory concentration responsible for 50% reduction in cell viability (IC_50_) was obtained after 24 h of treatment and determined by regression analysis of the data.

### 2.4. Ethical Statement

All animal care and experimental protocols were conducted following the guidelines of the institutional care and use committee (ethics for care and use of experimental animals of IBRAG/UERJ, CEUA-UERJ) under register number CEUA/053/2012.

### 2.5. Effects of LQB 123 on Bloodstream Trypomastigotes Viability

Bloodstream trypomastigote forms (Y strain) were obtained from infected albino Swiss mice at the parasitaemia peak by differential centrifugation and suspended in Dulbecco modified Eagle's medium (DMEM-Life Technology) supplemented with 10% FCS. 1 × 10^6^ cells/well were incubated at 37°C for 24 h in the presence of increasing concentrations of LQB 123 (150–1200 *μ*M). Viable trypomastigotes were counted in a Neubauer chamber. The inhibitory concentration responsible for 50% reduction in cell viability (IC_50_) was obtained after 24 h of treatment and determined by regression analysis of the data.

### 2.6. Effects of LQB 123 on Intracellular Amastigotes

For infection assays, murine peritoneal macrophages were isolated from the peritoneum of Swiss Webster mice with iced DMEM medium supplemented with 10% FCS. The concentration was adjusted to 1 × 10^6^ macrophages/well and incubated in at 37°C and 5% CO_2_ for 24 h. Nonadherent cells were removed; the cultures were washed with phosphate-buffered saline (PBS, 100 mM phosphate buffer and 150 mM NaCl, pH 7.4) and then infected with bloodstream trypomastigotes, Y strain (MOI 10 : 1). After 3 h of interaction, the noninternalized parasites were removed by washing with PBS. The cells were then incubated with or without LQB 123 (125 *μ*M–500 *μ*M) in fresh DMEM for 48 h. Cells were stained by quick Romanowsky-type stain (Panótico Rápido LB) and examined under light microscopy. The percentage of infection and the number of intracellular amastigotes were quantified using a light microscopy. The infection index was determined by the percentage of infected cells multiplied by the number of amastigotes per cell.

### 2.7. Toxicity to Mammalian Cells

Uninfected peritoneal macrophages (2.5 × 10^5^ cells/well) were treated with the LQB 123 for 48 h and their toxicity was evaluated by the alamarBlue® assay [21]. The reaction was analyzed at 570 nm detection, using the 600 nm absorbance as normalization value. The cell viability was not affected by the final DMSO concentration (data not shown).

### 2.8. *Salmonella*/Microsome Assay

Stationary growth cultures of histidine auxotrophic* Salmonella typhimurium* (2 × 10^9^ cells/mL) TA97, TA98, TA100, TA102, and TA1535 strains were obtained at 37°C in lysogenic broth (LB, 10 g/L tryptone; 5 g/L yeast extract; 10 g/L NaCl) containing 8 *μ*g/mL ampicillin and 2 *μ*g/mL tetracycline (only for TA102). All strains used in the present work were from our laboratory collection. Survival experiments were carried out without (−S9) and with (+S9) exogenous metabolization (Moltox™, Molecular Toxicology Inc., Boone, NC) in order to determine the nontoxic concentrations to be used in the subsequent studies (data not shown). Then, the Ames test was performed according to Maron and Ames [22]. DMSO was used as a negative control. The positive controls used for assays in the absence of S9 mix were 4-nitroquinoline-1-oxide (4-NQO), at 526 *μ*M and 263 *μ*M for TA97 and TA98, respectively; sodium azide (SA) at 769 *μ*M and 154 *μ*M for TA100 and TA1535, respectively; and mitomycin C (MMC), at 149 *μ*M; in the presence of S9 mix the positive controls were benzo[a]pyrene, at 396 *μ*M for TA102 and 2-aminoanthracene (2-AA), at 517 *μ*M for TA97, TA98, TA100, and TA1535. All chemicals were from Sigma Co.

### 2.9. Statistical Analysis

Statistical analysis was conducted with GraphPad Prism 5 software (GraphPad Software, Inc., San Diego, CA). Data were analyzed by one-way ANOVA, and differences between groups were assessed with Tukey's posttest. The level of significance was set at *p* < 0.05.

## 3. Results

### 3.1. LQB 123 Activity against* T. cruzi* Bloodstream and Metacyclic Trypomastigotes and Epimastigotes

The treatment with LQB-123 was effective against epimastigotes, bloodstream, and metacyclic trypomastigotes with IC50 in the range of 75–1200 *μ*M. For bloodstream trypomastigotes IC_50_/24 h it was 259.4 ± 6.1 *μ*M and for metacyclic trypomastigotes the IC_50_/24 h was 191.8 ± 10.5 *μ*M. The mammalian proliferative form of* T. cruzi* was more susceptible to LQB 123, with IC_50_/48 h values of 188.2 ± 47.5 *μ*M (Table 1). It is important to point out that* T. cruzi* is genetically classified into six intraspecies lineages, currently called discrete typing units (DTUs): TcI–VI [23]. This intraspecific diversity has been demonstrated by differences in morphology of blood forms, virulence, pathogenicity, immunological properties, infectivity in host cells, and susceptibility to chemotherapeutic agents [24]. The Dm28c clone belongs to DTU I, while Y strain belongs to DTU II. Despite that, the inhibitory concentrations for both strains or forms were similar.

### 3.2. LQB 123 Activity against* T. cruzi* Intracellular Amastigotes

**Figure 1:**
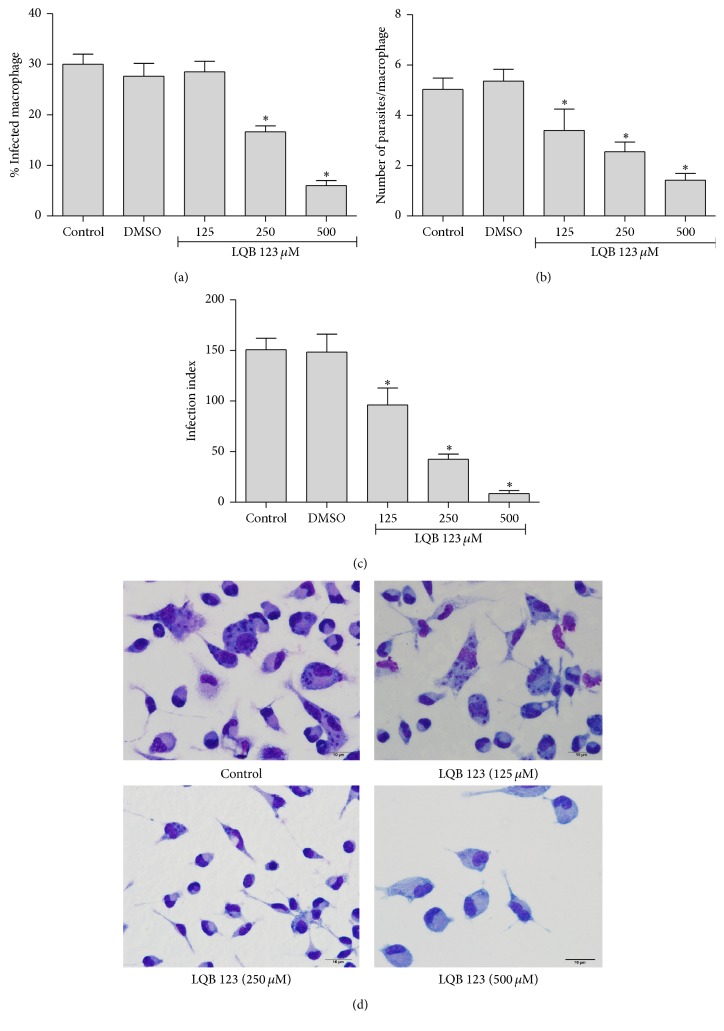
LQB 123 decreases* T. cruzi* infection. (a) The percentage of infection, (b) the number of parasites/macrophage, and (c) the infection index. The infection index was determined by the percentage of infected cells multiplied by the number of parasites per cell. (d) Light microscopy of* T. cruzi* infected macrophages after 3 days. The cells were stained by quick Romanowsky-type stain (Panótico Rápido LB) and examined under a light microscope at 100x magnification. Scale bars = 10 *μ*m. The data from DMSO represents the concentration present in the highest dose of the drug. Results are shown as mean ± SD; *∗* represents *p* < 0.05 in relation to the control group by ANOVA one-way test and Tukey's posttest. Data are representative of three independent experiments performed in triplicate.

Once more, LQB 123 exerted a strong inhibitory effect against another proliferative form of* T. cruzi*, the intracellular amastigotes. After 48 h, the compound decreased the number of infected cells in IC_50_ by 78.3%, the amastigotes inside each cell by 73.50%, and 50% of the infectivity index of amastigotes (188.2 *μ*M) compared to DMSO control (Figures 1(a), 1(b), and 1(c)).

### 3.3. Mammalian Toxicity Assay

The cytotoxicity assay was performed to evaluate the safety of the compound in noninfected peritoneal macrophages. The cells were exposed to different concentrations of LQB 123 for 48 h, and the concentration that was cytotoxic to 50% of the cells (CC_50_) was higher than 4000 *μ*M (data not shown). Interestingly, comparing the CC_50_ with the IC_50_/48 h, the nitrone exhibited higher Selective Index values comparing to metacyclic trypomastigotes (>21) or bloodstream trypomastigotes (>16), the relevant clinical forms of* T. cruzi* (Table 1), indicating that LQB 123 was more active against the parasite, showing no toxicity to uninfected macrophages in vitro.

### 3.4. *Salmonella*/Microsome Assay

The incubation with LQB 123 in the absence or in the presence of exogenous metabolization showed no mutagenic activity over the sensitive strains (mutagenic index ≤ 2). Also, a major reduction of the induced revertants was obtained for TA97, TA98, and strains even in the absence of metabolic activation (Table 2). Moreover, the highest concentration used (33000 *μ*M), corresponding to 127 times the IC_50_ for bloodstream trypomastigotes, led to a significant decrease in the survival of TA100 and TA1535 strains. However, these toxic effects were not observed in the presence of exogenous metabolization (except TA100 strains in the highest concentration) indicating that S9 mix can have a detoxifying effect on the compounds responsible for bacterial growth inhibition.

## 4. Discussion

After 107 years of its discovery, the chemotherapy of this disease is still controversial and unsatisfactory, provided that the only two available drugs are toxic, possibly carcinogenic, besides presenting low effectiveness against the chronic phase of the disease [25].

Despite that, efforts have been made in order to improve CD treatment using the available drug, benznidazole; however, so far, benznidazole did not result in a statistically significant improvement in cardiac clinical outcomes [12]. The fact that CD is a neglected tropical disease becomes very alarming if compared with the death rates and outcomes of nonneglected illnesses such as cancer. An extrapolation calculated using the deaths of BENEFIT clinical trial patients and those collected by WHO estimates that 200.000 people will die from Chagasic cardiomyopathy over the next five years [11]. Therefore, a next generation of new products is mandatory for the establishment of novel therapies for CD. In this scenario, organic molecules generically known as nitrones have been used for more than 30 years in the analytic chemistry and in biochemistry in order to detect and establish free radicals. The PBN (alpha-phenyl-tert-butyl nitrone) is among the most studied and used for this objective, being considered an antioxidant that scavenges a wide variety of free radical species and inhibits free radical generation and also demonstrating strong pharmacological activities in disease models related to the overproduction of mitochondrial ROS, as well as neurodegenerative illnesses, stroke, and Alzheimer [13].

Wen et al. [16] demonstrated an oral administration of PBN resulting in the preservation of cardiac functions, the increase of mitochondrial function, and oxidative stress decrease in chronically infected rats by* T. cruzi*. However, these effects were not able to decrease the inflammatory infiltrate, or the parasite persistency.

LQB 123 compound showed effectiveness against all biologic forms of* T. cruzi*. It is also possible to highlight the trypanocidal action against infective forms, metacyclic trypomastigotes, and bloodstream, with IC_50_/24 h 191.8 *μ*M and 259.4 *μ*M, respectively, and a higher susceptibility over the mammalian stage proliferative form (IC_50_/48 h 188.2 *μ*M) (Table 1). We also observed a LQB123 action in peritoneal macrophages cultures infected by this parasite. When 10 : 1 parasite/macrophage ratio was used, the LQB123 greatly diminished the percentage of infected macrophages, the intracellular amastigote number, and the decrease of infectivity index (Figure 1). Although presenting apparently elevated IC_50_, these nitrones had already been presented as highly tolerated molecules. In 2004, a clinical trial demonstrated the effectiveness of NXY-059 compound (another PBN derivative) in patients with ischemic stroke, even when concentrations higher than 260 *μ*M of the compound were administrated intravenously [26].

Our results also suggest that the modifications in the PBN that originated the derivate LQB 123 maintained a very low toxicity of the compound for mammalian cells. The alamarBlue viability assays showed no harmful effect in peritoneal macrophages cells, even in the presence of 4000 *μ*M of the nitrone, a concentration 21 times higher than the necessary to abolish the intracellular amastigote infection. Also, the LQB 123 compound presented greater selective action for the parasite highly infective trypomastigote forms with selectivity index higher than 16 for bloodstream trypomastigotes and higher than 21 for metacyclic trypomastigotes (Table 1). The marked selective effect of the LQB123 over intracellular amastigotes is also demonstrated in Figure 1(d), with 500 *μ*M of the compound totally abolishing the macrophage infection, showing once more no deleterious effect to the mammalian cell.

Following the method recommended by the Organization for Economic Cooperation and Development (OECD) TG471 [27], we applied the* Salmonella*/microsome assay. This assay is commonly employed as an initial screening to investigate the mutagenic potential of chemicals because there is a high predictive value for rodent carcinogenicity when a mutagenic response is obtained [28, 29]. LBQ 123 induced neither frameshift nor base pair substitutions at concentrations up to 2200 *μ*M, a dose that corresponds to 10 times the IC_50_ calculated for bloodstream trypomastigotes. The toxic effects were observed only at high concentrations (127 times the IC_50_ calculated for bloodstream trypomastigotes) and in the absence of exogenous metabolization, strongly indicating that S9 mix probably has a detoxifying effect of the nitrone structure, responsible for the inhibition of bacterial growth (Table 2).

Another possible chemotherapy applicability of LQB 123 is further enhanced because the benznidazole mutagenic activity was tested by Ames assay [30]. The maximum mutagenic rate of benznidazole was obtained at 100 micrograms per plate (20 times IC_50_ calculated for bloodstream trypomastigotes). This concentration when applied to LQB 123 presents no mutagenic effect.

Thus, it is plausible to suggest that the LQB 123 molecule retained some of the chemical properties of PBN, such as the low toxicity and a prominent trypanocidal effect in vitro. It is important to point out that the the beneficial effect of PBN in vivo against CD was a consequence of its free radical scavenging properties; however, recent reports have shown that the antileukemic properties of the PBN derivate, the O-geranylated nitrone (LQB-278), were not free radical related but a consequence of cell cycle regulation by the inhibition of p21 expression [19]. Therefore, the antioxidant property may not be the only mechanism of action of these molecules. Thus, further experiments using biochemical and molecular approaches are needed to better characterize the mechanism of action of the LQB123. Several well-known examples of different modes of action of closely related analogues can be found in medicinal chemistry. So, gradually PBN or LQB 123 changes from a more specific trypanocidal compound effect are necessary.

Finally, it is important to establish that a desired compound should have trypanocidal activity without inducing toxic effects to cells at concentrations that can be achieved in vitro. The relative effectiveness of an investigational product in inhibiting parasite survival compared to inducing cell death and the therapeutic or selectivity index has to be considered. Thus, the selection of new trypanocidal compounds should take into account a high selectivity index giving maximum trypanocidal activity with minimal cell toxicity.

Also, the new therapies to be established should include new pediatric formulations of the available drug, benznidazole, and new benznidazole treatment regimens (including combination therapies), as well as a new therapeutic vaccine linked to benznidazole chemotherapy [31].

## 5. Conclusions

Taken together, these results broaden new perspectives for the Chagas disease treatment once LQB 123, a novel nitrone derivate, significantly decreased mammalian cells infections by* T. cruzi*. Furthermore, the low toxicity of LQB 123 against the vertebrate cells, proven in this work, is a very important result that justifies future animal experimentation, as well as the testing of new PBN analogues.

## Figures and Tables

**Table 1:** LQB 123 effect upon *Trypanosoma cruzi* forms.

Parasite forms	IC_50_ (*μ*M)	Time (h)	SI
Epimastigotes (Y strain)	255.15 ± 32.6	24	>15
Metacyclic trypomastigotes (Dm28c)	191.8 ± 10.5	24	>21
Bloodstream trypomastigotes (Y strain)	259.4 ± 6.1	24	>16
Intracellular amastigotes (Y strain)	188.2 ± 47.5	48	>21

The trypanocidal activity was expressed as the IC_50_, corresponding to the concentration that leads to 50% parasite lysis.

CC_50_ > 4000 *μ*M (drug concentration which reduced 50% of peritoneal macrophage viability).

SI (selectivity index) = CC_50_/IC_50_.

**Table 2:** Number of *Salmonella typhimurium* TA97, TA198, TA100, TA1535, and TA102 revertant colonies per plate in the presence of the PBN derivate following the preincubation procedure of the *Salmonella*/microsome assay in the absence (−) and in the presence (+) of exogenous metabolizing system from rat liver S9-mix.

Strain	Dose (*µ*M)	−S9		+S9	
		Mean ± SD^a^	MI^b^	Mean ± SD^a^	MI^b^
TA97	0	112 ± 9	1.0	166 ± 19	1.0
	22	114 ± 20	1.0	192 ± 15	1.2
	220	92 ± 22	0.8	192 ± 18	1.2
	2200	97 ± 25	0.9	183 ± 11	1.1
	22000	11 ± 2^*∗*^	0.1^*∗*^	183 ± 18	1.1
	33000	1 ± 0^*∗*^	0.0^*∗*^	155 ± 11	0.9
	PC	1304 ± 226^*∗*^	**12.0**	1488 ± 139^*∗*^	**9.0**

TA98	0	24 ± 9	1.0	19 ± 1	1.0
	22	31 ± 2	1.3	22 ± 6	1.2
	220	29 ± 1	1.2	18 ± 7	1.0
	2200	25 ± 4	1.0	19 ± 1	1.0
	22000	8 ± 3^*∗*^	0.3^*∗*^	18 ± 9	1.0
	33000	7 ± 2^*∗*^	0.3^*∗*^	20 ± 2	1.1
	PC	613 ± 32^*∗*^	**26.0**	291 ± 36^*∗*^	**16.0**

TA100	0	201 ± 35	1.0	272 ± 45	1.0
	22	221 ± 28	1.1	242 ± 41	0.9
	220	205 ± 5	1.0	274 ± 35	1.0
	2200	154 ± 19	0.8	266 ± 38	1.0
	22000	134 ± 10^*∗*^	0.7^*∗*^	226 ± 33	0.8
	33000	52 ± 0^*∗*^	0.3^*∗*^	215 ± 25	0.8
	PC	637 ± 52^*∗*^	**3.2**	1257 ± 107^*∗*^	**4.6**

TA1535	0	13 ± 0	1.0	17 ± 3	1.0
	22	15 ± 2	1.2	17 ± 3	1.0
	220	14 ± 2	1.1	15 ± 3	0.9
	2200	11 ± 3	0.8	12 ± 1	0.7
	22000	11 ± 2	0.8	12 ± 1	0.7
	33000	12 ± 2	0.9	12 ± 1	0.7
	PC	615 ± 83^*∗*^	**47.3**	168 ± 18^*∗*^	**10.1**

TA102	0	433 ± 10	1.0	202 ± 12	1.0
	22	449 ± 30	1.0	214 ± 16	1.1
	220	427 ± 29	1.0	218 ± 8	1.1
	2200	409 ± 45	0.9	201 ± 11	1.0
	22000	534 ± 67	1.2	181 ± 26	0.9
	33000	533 ± 15	1.2	152 ± 7	0.8
	PC	1638 ± 201^*∗*^	**3.8**	1136 ± 78^*∗*^	**5.6**

^a^Number of revertant colonies per plate: mean and standard deviation (SD) values of three replicates. ^b^MI: mutagenicity index: ratio of the number of revertant colonies induced by the samples/spontaneous number by the negative control. Positive mutagenicity (MI > 2) is indicated in bold. The sample vehicle (DMSO) was tested as a negative control. The doses of the positive controls (PC) in assays without S9: 526 *μ*M and 263 *μ*M of 4-NQO (TA97 and TA98, resp.), 769 *μ*M and 154 *μ*M of AS (TA100 and TA1535, resp.), and 149 *μ*M of MMS (TA102). In assays with S9: 396 *μ*M of B[*α*]P (TA102) and 517 *μ*M of 2-AA (for others strains), statistically significant differences (^*∗*^*p* < 0.05) relative to the negative control by ANOVA and Tukey's test are indicated.
